# Bone morphogenetic protein-2: a potential regulator in scleral remodeling

**Published:** 2008-12-18

**Authors:** Jianmin Hu, Dongmei Cui, Xiao Yang, Shaowei Wang, Shoulong Hu, Chuanxu Li, Junwen Zeng

**Affiliations:** State Key Laboratory of Ophthalmology, Zhongshan Ophthalmic Center, Sun Yat-sen University, Guangzhou, P. R. China

## Abstract

**Purpose:**

Bone morphogenetic protein 2 (BMP-2) is a member of the main subgroup of bone morphogenetic proteins within the transforming growth factor-β superfamily. BMP-2 is involved in numerous cellular functions including development, cell proliferation, apoptosis, and extracellular matrix synthesis. We examined BMP-2 expression in human scleral fibroblasts (HSF) and assessed the effects of recombinant human BMP-2 (rhBMP-2) on HSF proliferation, matrix metalloproteinase-2 (MMP-2), and tissue inhibitor of metalloproteinase-2 (TIMP-2).

**Methods:**

We used confocal fluorescence microscopy (CFM) to study BMP-2 distribution in HSF cells and frozen human scleral sections. The influence of rhBMP-2 on cell proliferation at different concentrations (0 ng/ml, 1 ng/ml, 10 ng/ml, and 100 ng/ml) was evaluated by the 3-(4, 5-dimethylthiazol-2-yl)-2, 5-diphenyltetrazolium bromide (MTT) assay. The effects of rhBMP-2 on the cell cycle were investigated with flow cytometric analysis. Reverse transcription polymerase chain reaction (RT–PCR) and enzyme-linked immunosorbent assay (ELISA) were used to examine *MMP-2* and *TIMP-2* mRNAs and secreted proteins in HSF that were incubated with rhBMP-2.

**Results:**

BMP-2 protein expression from human sclera was confirmed by CFM. Cell proliferation was significantly increased with 100 ng/ml rhBMP-2 in a time-dependent manner (p<0.05). The HSF cell cycle moved to the S and S+G_2_M phases after rhBMP-2 stimulation at 100 ng/ml compared to normal cells (p<0.05). *TIMP-2* mRNA levels were significantly increased in HSF incubated for 24 h with 100 ng/ml rhBMP-2 (p<0.01). A 48 h incubation with 10 ng/ml or 100 ng/ml rhBMP-2 resulted in significantly increased *TIMP-2* mRNA and protein expression and significantly decreased *MMP-2* mRNA expression (p<0.01) while MMP-2 protein expression significantly decreased at 100 ng/ml rhBMP-2 (p<0.01).

**Conclusions:**

Human sclera fibroblasts expressed BMP-2, which promoted cell proliferation, and elicited changes in MMP-2 and TIMP-2, might influence extracellular matrix synthesis.

## Introduction

Scleral fibroblasts are involved in scleral remodeling, which occurs during axial elongation in myopia [1-3]. Many aspects of scleral extracellular matrix remodeling and fibroblast proliferation are regulated by specific growth factors including transforming growth factor-β (TGF-β), insulin-like growth factors I and II (IGF-I and IGF-II), and basic fibroblast growth factor (bFGF) [4-6]. The matrix metalloproteinase-2 (MMP-2) and tissue inhibitor of metalloproteinase-2 (TIMP-2) proteoglycans are involved in scleral extracellular matrix remodeling events associated with eye growth under both normal and myopic conditions [7,8].

Bone morphogenetic proteins (BMPs) are subgroups from the transforming growth factor-β (TGF-β) superfamily and have numerous cellular functions including development, morphogenesis, cell proliferation, apoptosis, and extracellular matrix synthesis [9]. Recent research suggests that BMPs have multiple body functions and are frequently designated “body morphogenetic proteins” [10]. BMP-2 is a bone morphogenetic protein and is also a potent osteoinductive cytokine. It induces bone and cartilage formation, controls fibroblast apoptosis, and regulates extracellular matrix synthesis in many tissues [11,12]. Additionally, BMP-2 expression has been detected in both adult and embryonic tissues of the cornea, the trabecular meshwork, the optic nerve head, the retina, and the conjunctiva [13-15]. Recent studies have suggested that BMP-2 is possibly involved in the pathophysiology of several ocular diseases [14-16].

Previous studies have not confirmed the presence of BMPs in the human sclera. We examined human scleral fibroblasts (HSF) for BMP-2 and examined the effects of recombinant human BMP-2 on HSF proliferation and extracellular matrix synthesis in this study.

## Methods

### Tissue source

This study was approved by the Ethics Committee of Sun Yat-sen University (Guangzhou, China) and complied with the tenets of the Declaration of Helsinki for biomedical research involving human subjects. Healthy adult human eyes (n=4) from donors (age range 18–23 years) were obtained from the Eye Bank of the Zhongshan Ophthalmic Center (Sun Yat-sen University). The specimens were numbered 1-4.

### Sample sclera preparation

The anterior segments, vitreous bodies, choroids, and retinas were removed from specimens 1 and 2. The posterior sclera was cut into 5×5 mm^2^ pieces, embedded with optimum cutting temperature compound (Sigma, St. Louis, MO), and cut into 5 μm sections at −20 °C. Sections were subsequently tiled onto slides (Corning Ltd, Tokyo, Japan), fixed with cool acetone for 15 min, air-dried, and kept frozen at −20 °C until use.

### Human scleral fibroblast isolation, culture, and identification

Specimens 3 and 4 were washed immediately in Hank's balanced salt solution (HBSS, Gibco, Grand Island, NY) with penicillin (200 ug/ml; Invitrogen Corp, Carlsbad, CA) and gentamicin sulfate (400 μg/ml; Invitrogen Corp, Carlsbad, CA). All eye contents were removed except the sclera. The sclera were trimmed into 1×1 mm pieces, placed in 25 mm^2^ plastic culture bottles in Dulbecco’s modified Eagle's medium (DMEM, Gibco) with 1X antibiotic/antimycotic (Invitrogen Corp), and 10% fetal bovine serum (FBS, Gibco), and incubated at 37 °C in a humidified incubator containing 5% CO_2_. The growth medium was changed every three days, and upon achievement of a heavy primary monolayer, the cells were trypsinized for 2 min at room temperature in 0.25% trypsin/EDTA solution in phosphate buffered saline (PBS, Gibco). Cells were subcultured at a split ratio of 1:3 in a 25 mm^2^ plastic bottle (Corning Ltd), and the third fibroblast passage was used for this experiment. The fibroblasts were grown on coverslips in six well plates (Corning Ltd) to 70%-80% confluence. The cells were washed with PBS three times, fixed with cool acetone for 15 min, air-dried, and kept frozen at −20 °C until use. HSF morphology was observed with light microscopy. The purity of fibroblast cell cultures was confirmed by staining for vimentin and stain resistance for cytokeratin, desmin, and S-100 in the indirect immunofluorescence procedure as previously described [17].

### Indirect immunofluorescence

The slides were washed three times with PBS, covered with 10% normal goat serum (Boster Biological Technology, Wuhan, China) diluted in PBS, and incubated for 20 min at 37 °C. The slides were incubated at 4 °C overnight with primary antibodies (anti-BMP-2; Boster Biological Technology) diluted to 1:500 in PBS. Cells were incubated in PBS without primary antibodies as a negative control. The antibody-treated and negative control sample slides were washed with PBS and exposed to fluorescein isothiocyanate-conjugated (FITC) goat anti-rabbit IgG antibodies (Boster Biological Technology) diluted at 1:50 in PBS at 37 °C for 30 min. The slides were washed with PBS three times and stained with propidium iodide (Sigma) and Hoechst 33342 (Sigma) to stain the cell nuclei. Immunofluorescent images were taken with a confocal microscope (LSM 510 META, Carl Zeiss, Jena, Germany).

### Assay of BMP-2 on human sclera fibroblast proliferation and cell cycles

The influence of cell proliferation was evaluated by the 3-(4, 5-dimethylthiazol-2-yl)-2, 5-diphenyltetrazolium bromide (MTT, Gibco) assay. Recombinant human BMP-2 (rhBMP-2; Peprotech Inc., Rocky Hill, NJ) was reconstituted in water containing bovine serum albumin (BSA; Sigma) to a concentration of 0.1 mg/ml, and this solution was diluted into DMEM to different concentrations and stored at 4 °C for immediate use or frozen in aliquots at −20 °C. The first passage cells were plated in standard flat-bottomed 96 well plates (Corning Ltd) with DMEM in 10% FBS at a 4,000 cells/cm^2^ density. Before treated with rhBMP-2 or control BSA, the medium was changed to DMEM without FBS for 24 h after initial plating. The culture media were replaced by various media containing test agents after an incubation of 24 h. Cells were incubated at 37 °C for one to seven successive days, and rhBMP-2 and control samples were replaced with fresh agents daily. Cells were washed twice with 10 mmol/l PBS (pH 7.2) and incubated with 0.5 mg/ml MTT for the last 4 h before the end of the incubation. The medium was decanted, formazan salts were dissolved with 200 µl dimethyl sulfoxide (DMSO, Sigma), and the absorbance was determined at 490 nm using an enzyme linked immunosorbent assay (ELISA) reader (BIO-TEK Instruments, Winooski, VT).

The rhBMP-2 effects on the cell cycle were investigated by flow cytometry. The HSF were stimulated with 100 ng/ml rhBMP-2, and the control group was trypsinized. Cells were centrifuged for 5 min at 600x g, washed with PBS, and fixed with ice-cold 70% (v/v) ethanol. Cells were washed twice with PBS, treated with 25 units RNase (Sigma) at room temperature for 10 min and finally resuspended in PBS with 50 μg/ml propidium iodide. DNA fluorescence was measured with a FACSCalibur Flow Cytometry System (Becton-Dickinson immunocytometer systems, San Jose, CA). The cell cycle distribution by FACS data was analyzed using the ModFit LT software (Verity Software House, Topsham, ME).

### Reverse transcription polymerase chain reaction for mRNA expression of *MMP-2* and *TIMP-2*

The *MMP-2* and *TIMP-2* mRNA expression values were determined with reverse transcription polymerase chain reaction (RT–PCR) with *β-actin* amplification as a control. Human sclera fibroblasts were seeded in 75 cm^2^ Nunc culture bottles at a density of 1×10^6^ cells per bottle and cultured for 24 h.

Various concentrations of rhBMP-2 (0 ng/ml, 1 ng/ml, 10 ng/ml, and 100 ng/ml) were added to samples after synchronization of HSF cells in DMEM without FBS for 24 h as described in the MTT assay. The control group was incubated without the addition of drugs. Total RNA was extracted from cells with TRIzol reagent (Invitrogen) after 12 h, 24 h, and 48 h incubation, and cDNA was synthesized with 5 µg total RNA, 1 µl random primer, 2 µl dNTPs, and 200 U MMLV Reverse Transcriptase (Promega, Madison, WI) at 37 °C for 1 h. *MMP-2* and *TIMP-2* mRNAs were detected by RT–PCR. The nucleotide sequences of the primers used in the experiments and the GenBank accession number of the underlying sequences are denoted in Table 1. Amplification was as follows 30 denaturing cycles at 94 °C for 45 s, annealing cycles at 59 °C for 30 s, and an extension cycle at 72 °C for 60 s. The reaction mixture (25 µl) contained 3 µl cDNA, 25 pmol of each primer, 0.25 mmol dNTPs, and 2 U of Taq DNA polymerase (Promega). PCR products were analyzed with 1% agarose gel electrophoresis and visualized with ethidium bromide staining. PCR product fragment sizes produced were 657 bp (*MMP-2*), 305 bp (*TIMP-2*), and 513 bp (*β-actin*). All RT–PCR products were compared to β-actin cDNA products from corresponding samples, and all band intensities were evaluated by densitometry.

**Table 1:** Primer sequences used in polymerase chain reaction.

**Name**	**GenBank accession number**	**Upstream primer**	**Downstream primer**	**Size (bp)**
*MMP-2*	NM_001127891	ATGCGGAAGCCACGCTGCG	AGCGGTAGCCATCCGTGCG	657
*TIMP-2*	NM_003255	TACCAGATGGGCTGCGAGTG	TCCAGGAAGGGATGTCAGAG	305
*β-actin*	NM_001101	CCTAGAAGCATTGCGTGG	GAGCTACGAGCTGCCTGACG	513

### Enzyme-linked immunosorbent assay

MMP-2 and TIMP-2 proteins secreted from primary HSF cells were incubated with 0 ng/ml, 1 ng/ml, 10 ng/ml, and 100 ng/ml rhBMP-2 after 12 h, 24 h, and 48 h. Proteins were assayed with commercially available enzyme-linked immunosorbent assay (ELISA) kits (USCN Life Science & Technology Company, Double Lake, MO). Conditioned media from the primary HSF cell culture were used in a 1:100 dilution to ensure that MMP-2 and TIMP-2 levels fell within the standard assay ranges. Lower detection limits were 0.390 ng/ml (MMP-2) and 0.312 ng/ml (TIMP-2). There was no significant cross-reactivity or interference, and the intra-assay and inter-assay variability was less than 5% according to the manufacturer's description. All samples were assayed, and the 450 nm optical density (OD) was determined with an ELISA reader. Human MMP-2 and TIMP-2 from conditioned media were quantified in duplicate with double-sandwich ELISA kits according to the manufacturer’s protocol. The levels of secreted MMP-2 and TIMP-2 were expressed as ng/ml values.

### Statistical analysis

Data were expressed as the mean±standard deviation (SD). The Student’s* t*-test was performed for statistical analysis of cytometry between the experiment and control groups. Groups were compared by using a one-way analysis of variance (ANOVA) with a Tukey post hoc test where p<0.05 indicated a statistically significant difference. All analyses were performed with software (SPSS version 12.0; SPSS, Chicago, IL).

## Results

### BMP-2 expression in scleral cytoplasm and tissues

**Figure 1:**
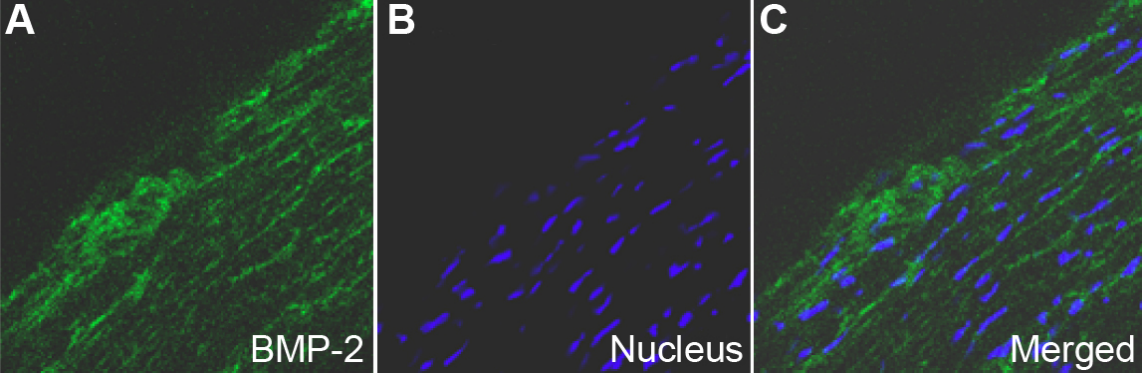
Distribution of BMP-2 in human sclera by indirect immunofluorescence. FITC marked the secondary antibody (green; **A**) and Hoechst33342 dyed the nucleus (blue; **B**). The first (**A**) and second images (**B**) are combined to form the third image shown (**C**). BMP-2 is localized in the cytoplasm of HSF and extracellular matrices (**A**-**C**). Magnification: 400X.

**Figure 2:**
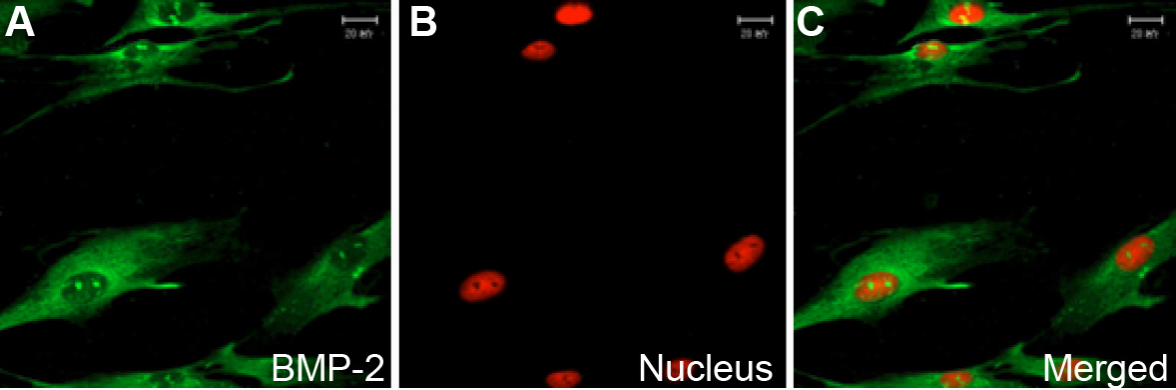
Distribution of BMP-2 in human sclera fibroblasts in vitro using indirect immunofluorescence. FITC marked the secondary antibody (green; **A**), and PI dyed the nucleus (red; **B**). The first (**A**) and second images (**B**) are combined to form the third image shown (**C**). BMP-2 is localized in the cytoplasm and weakly in the nucleus of HSF (**A**-**C**). Magnification: 400X.

In vivo, BMP-2 levels were uniformly expressed in the HSF cytoplasm and extracellular matrices of scleral tissue (Figure 1). In vitro, BMP-2 was expressed mainly in the cytoplasm of primary cultured HSF and weak in the nuclei (Figure 2).

### Affects of rhBMP-2 on cell proliferation and the cell cycle in rhBMP-2 culture

**Figure 3:**
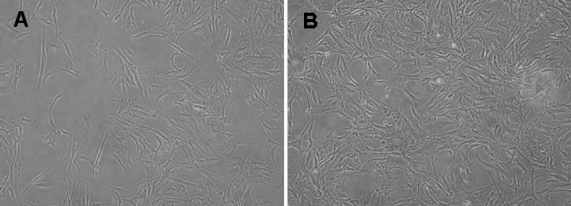
The changed pattern of human sclera fibroblasts incubated with rhBMP-2. The HSFs showed only a sparse and vortex pattern before incubation (**A**) whereas incubation with 100 ng/ml rhBMP-2 changed the HSFs into the intensive and polygonal cells (**B**). Inverted phase contrast microscope, original magnification 100X.

**Figure 4:**
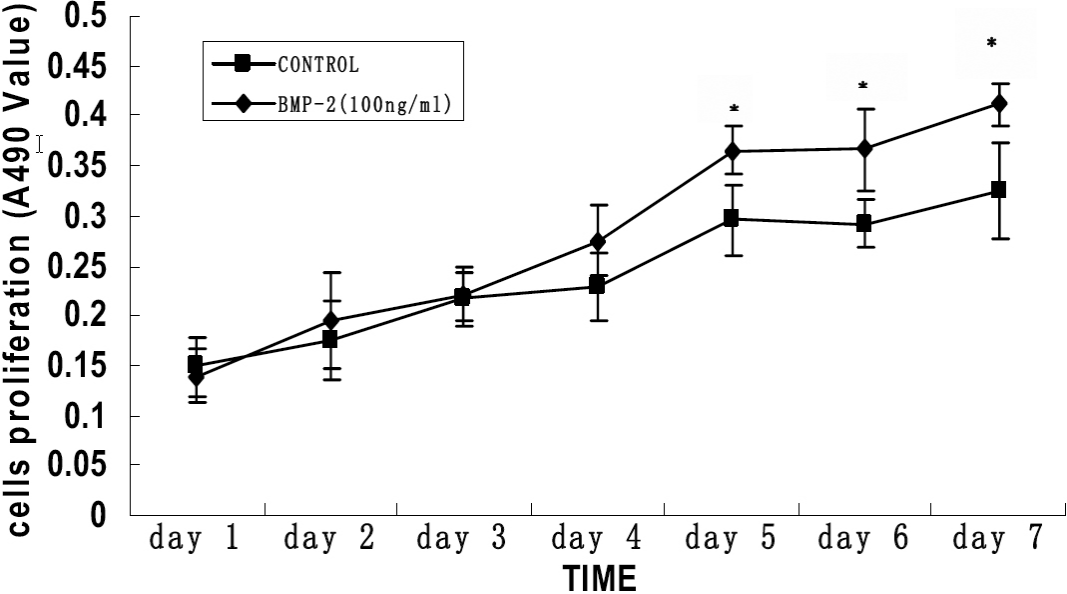
Time-dependent effects of rhBMP-2 on the cell proliferation of primary cultured human sclera fibroblasts. Cell proliferation was determined by MTT assay. The optical density value at A490 and the mean±SD is provided for each time point (n=8). The cells were incubated with no rhBMP-2 or 100 ng/ml rhBMP-2 for seven days. A significant difference was seen in cell proliferation between 100 ng/ml rhBMP-2 treatment and control after four days. The asterisk indicates that p<0.05 when compared with the control without rhBMP-2.

The HSF migrated from pieces of sclera tissue and populated the surroundings; cells exhibited a uniform, fusiform shape. Cell growth was arranged in a vortex pattern (Figure 3A). The HSF pattern in cells exposed to various concentrations of rhBMP-2 was polygonal (Figure 3B). HSF were exposed to various concentrations of rhBMP-2 (1 ng/ml, 10 ng/ml, and 100 ng/ml), and cells and controls were incubated for another one to seven days. HSF proliferation in the presence of rhBMP-2 (100 ng/ml) was significantly higher on day 5 than those of different rhBMP-2 concentrations or controls (p<0.05; Table 2). The maximum stimulatory effects on cell proliferation were achieved on day 5 through day 7 when rhBMP-2 was at a 100 ng/ml concentration, although the increase was not significant on day 1 through day 4 at the same concentration (Figure 4). Stimulatory effects were not seen at other concentrations.

**Table 2:** The effect of rhBMP-2 on HSF proliferation at different concentrations after five days.

**Group**	**Concentration**	**A value (X±s)**
1	0.00 ng/ml	0.2911±0.039
2	0.1 ng/ml	0.2996±0.056
3	1 ng/ml	0.2953±0.075
4	10 ng/ml	0.3023±0.032
5	100 ng/ml	0.3721±03042*

HSF proliferation in the presence of rhBMP-2 (100ng/ml) was significantly higher on day 5 than those of different rhBMP-2 concentrations or controls (p<0.05). Absorbance value (A value) at a wavelength of 490 nm and the mean±SD (X±s) is provided for each point (n=8) on day 5. An asterisk indicates p<0.05 when compared to the control group.

Flow cytometry analysis results revealed that the HSF percentages were increased significantly (p<0.05) in the S phase and decreased significantly (p<0.05) in the G_l_ phase when the cells were stimulated with 100 ng/ml BMP-2**. **Additionally, the Proliferation Index (Pr I(S+G_2_M)) was significantly higher in experiment group than that in control group (p<0.05; Table 3).

**Table 3:** Results of cytometry analysis.

**Group**	**G_1_**	**S**	**G_2_M**	**Pr I (S+G_2_M)**
Control	84.3%	6.4%	9.3%	15.7%
Experiment	61.3%*	18.9%*	19.8%	38.7%*

The HSF percentage was increased in the S phase and decreased in the G_l_ phase when the cells were stimulated with 100 ng/ml BMP-2**. **Additionally, the Proliferation Index (Pr I(S+G_2_M)) was also significantly increased. Proliferation index (Pr I) indicates the vability of cell proliferation, Pr I =(S+G_2_M)/(G_0_/G_1_+S+G_2_M). An asterisk indicates p<0.05 when compared to the control group.

### Changes in *MMP-2* and *TIMP-2* mRNA levels in rhBMP-2 culture

**Figure 5:**
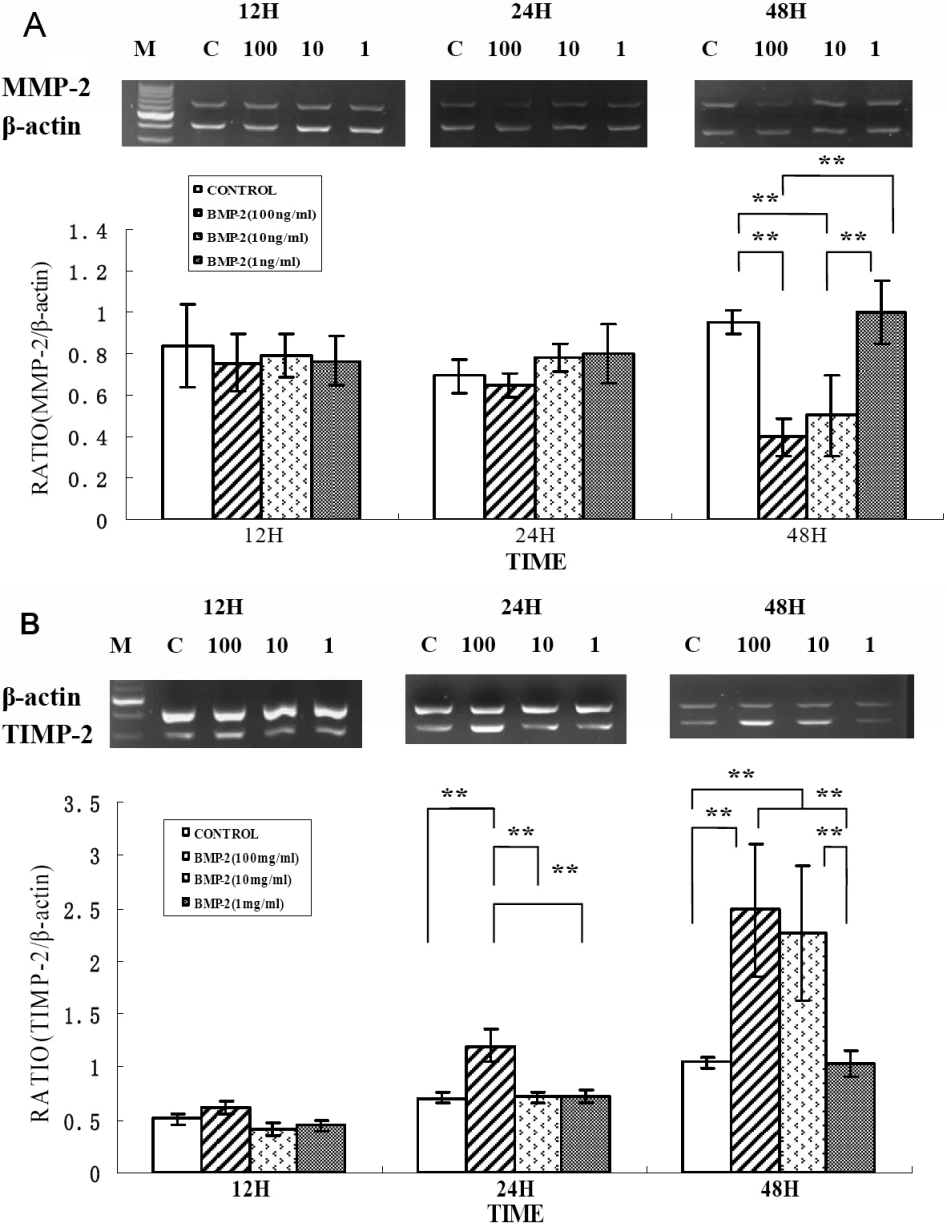
Effect of rhBMP-2 on mRNA expression levels of MMP-2 and TIMP-2 in human sclera fibroblasts. Ethidium-bromide agarose gels indicated the level of *β-actin* message relative to *MMP-2* (**A**) and *TIMP-2* (**B**) levels from total RNA. Bar graphs revealed changes in mRNA expression (mean±standard error of the mean) where values were normalized to *β-actin* values and expression was stated as a ratio of optical density. The double asterisk means that the semi-quantitative RT-PCR revealed significant changes for *MMP-2* and *TIMP-2* with varying concentrations of rhBMP-2 (p<0.01).

Relative *MMP-*2 and *TIMP-2* mRNA levels were markedly changed in BMP-2 cultures compared to *β-actin* mRNA levels in the negative controls. No differences were noted in the *MMP-2* and *TIMP-2* mRNA levels in HSF control samples and in samples incubated with rhBMP-2 at varying concentrations after 12 h. At 24 h, there was no significant change in the *MMP-2* mRNA level in any of the samples (all p>0.05). *TIMP-2* mRNA expression in HSF cells was significantly increased in samples incubated with 100 ng/ml rhBMP-2 (p=0.001). Significantly increased *TIMP-2* mRNA expression and decreased *MMP-2* mRNA expression were noted in HSF cells incubated with 100 ng/ml (both p=0.001) or 10 ng/ml (both p=0.003) rhBMP-2 for 48 h (Figure 5).

### Changes in MMP-2 and TIMP-2 protein levels in rhBMP-2 culture

**Figure 6:**
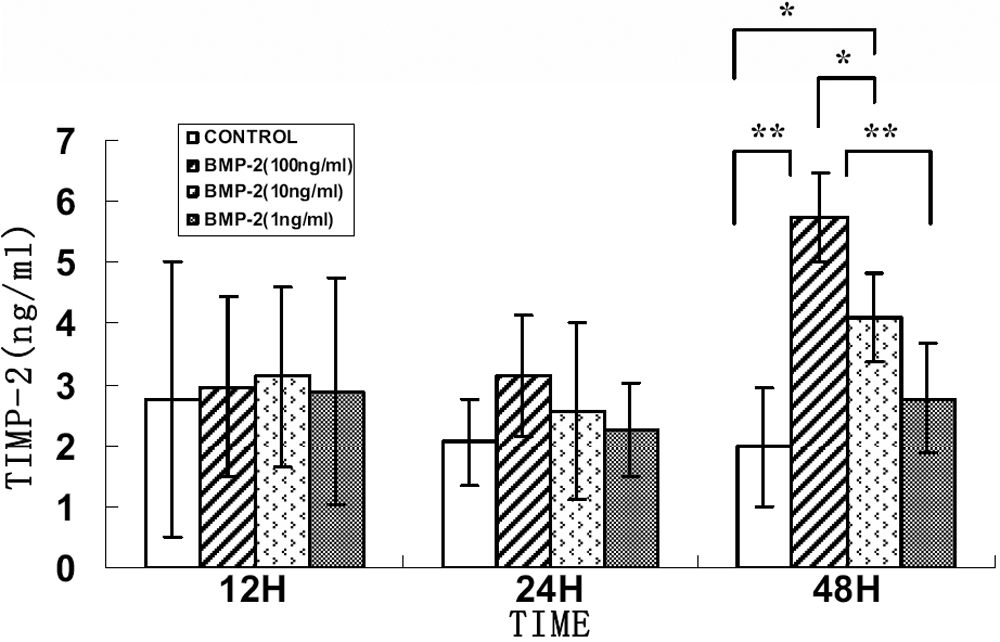
Effect of BMP-2 on TIMP-2 protein secreted from human sclera fibroblasts into culture medium. Cells were treated with 0, 1, 10, and 100 ng/ml rhBMP-2 for 12, 24, and 48 h. There were no significant differences in secreted protein levels of TIMP-2 in the HSF incubated with different rhBMP-2 concentrations after 24 h. The TIMP-2 protein levels were significantly increased following treatment with 100 ng/ml rhBMP-2 after 48 h (p< 0.01) and 10 ng/ml rhBMP-2 after 48 h (P < 0.05). Data were represented as the mean±SEM. Data were from three independent experiments. Each sample was assayed in duplicate. An asterisk indicates a p<0.05 compared to control values, and a double asterisk indicates a p< 0.01 compared to control values.

**Figure 7:**
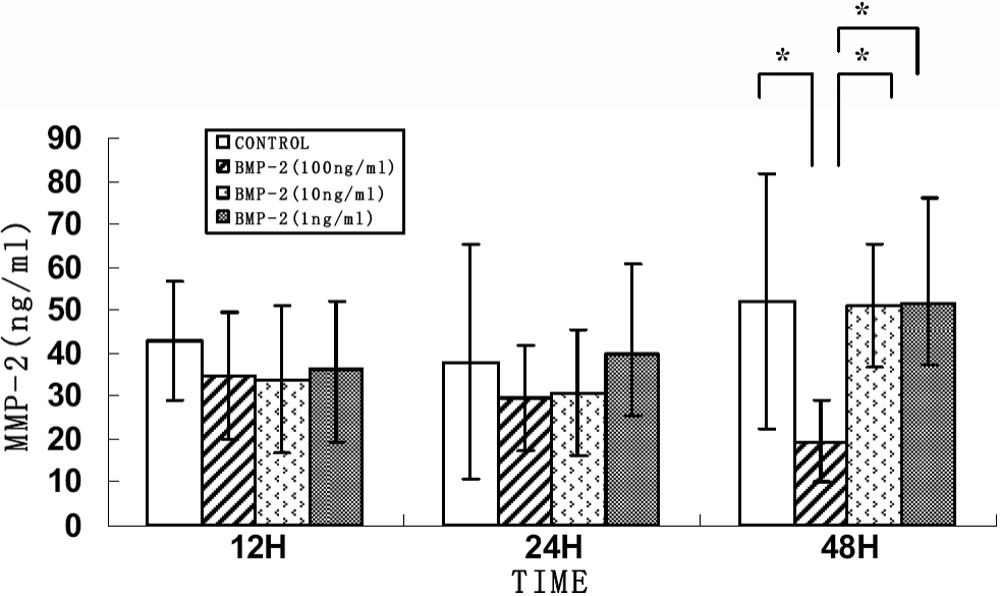
Effect of BMP-2 on MMP-2 protein secretion from human sclera fibroblasts into culture medium. Cells were treated with 0, 1, 10, and 100 ng/ml rhBMP-2 for 12, 24, and 48 h. There were no significant differences in secreted protein levels of MMP-2 in the HSF incubated with different rhBMP-2 concentrations after 24 h. The MMP-2 protein levels were significantly decreased following treatment of HSF cells with 100 ng/ml rhBMP-2 after 48 h (p<0.05). Data were represented as the mean±SEM. Data were from three independent experiments. Each sample was assayed in duplicate. An asterisk indicates a p<0.05 compared to control values.

There were no significant changes in secreted levels of MMP-2 and TIMP-2 proteins in HSF incubated with different rhBMP-2 concentrations after 24 h. The TIMP-2 protein levels were significantly increased following treatment of HSF cells with 100 ng/ml rhBMP-2 after 48 h (p=0.001) and 10 ng/ml rhBMP-2 after 48 h (p=0.038) as seen in Figure 6. MMP activities are regulated by TIMPs, and therefore, the MMP-2 protein level at 100 ng/ml rhBMP-2 was decreased significantly (p=0.044) as seen in Figure 7.

## Discussion

In addition to previous research that revealed BMP-7 expression in the rat sclera [18], we provided evidence of BMP-2 distribution in HSF and human sclera in this study. Our study revealed that BMP-2 can be localized in HSF to the nucleus and the cytoplasm. BMP-2 is a 26 kDa protein composed of two identical 114 amino acid polypeptide chains (monomers) linked by a single disulfide bond.

In our study, rhBMP-2 significantly promoted HSF cell proliferation in vitro only at a concentration of 100 ng/ml, and in a time-dependent manner; it also increased the HSF percentage in the S and S+G_2_M phases and decreased the HSF percentage in the G_l_ phase. This suggests that rhBMP-2 stimulated mitosis of *HSF* DNA and promoted cell proliferation as a result. BMP-2 is expressed in the human cornea and is considered a heparin-binding cytokine that can stimulate positive chemotaxis and modulate corneal fibroblast apoptosis [19]. The alterations in the scleral fibroblast population are involved in the modulation of scleral matrix turnover during myopia development [20]. Accordingly, BMP-2 is an important growth factor influence on HSF and is possibly involved in the growth of human sclera.

Significantly increased expression of TIMP-2 and decreased expression of MMP-2 in HSF after incubation with rhBMP-2 were observed in our present study. These results are similar to those in a recent study demonstrating the influence of rhBMP-2 on *MMP-2* and *TIMP-2* mRNA expression in bone regeneration [21]. Human scleral fibroblasts express MMP-2 (gelatinase A, 72-kDa gelatinase, Type IV collagenase) [22] and TIMP-2 [23]. MMP-2 is a protein initially secreted as a pro-enzyme (ProMMP-2) and converted to an active enzyme after cleavage (ActiveMMP-2). TIMP-2 inhibits MMP-2 activation and MMP-2 activity by blocking further cleavage of the pro-form active site cleft and by blocking the catalytic domain of both active MMP-2 and MMP-14 [24-26]. Scleral remodeling is a dynamic process that involves continual synthesis and degradation of the extracellular matrix. Degradation of scleral connective tissue during remodeling is partially regulated by the balance between MMPs and TIMPs [27,28]. Elevation of MMP activity in the sclera by cytokines associated with HSF has been implicated as a potential mechanism of tissue destruction in myopia [28,29]. All these suggested that BMP-2 may act as a cytokine in the control of scleral extracellular matrix remodeling in myopia progression.

The human sclera is a dynamic tissue and is capable of rapid responses to changes in the visual environment. It alters ocular size and refraction, and the HSF proliferation and scleral extracellular matrix remodeling events can result in dramatic and immediate changes in biomechanical properties of the sclera and subsequent changes in ocular length [30-33]. A gene microarray analysis was used to identify gene expression changes in human scleral fibroblasts in response to mechanical loads and possible mechanisms of scleral remodeling in the development of myopia and showed that mechanical stresses induced *BMP-2* mRNA expression after 30 min and 24 h [30]. We detected BMP-2 protein in HSF, and our data showed rhBMP-2 promotion of cell proliferation and altered the expression of MMP-2 and TIMP-2 in HSF. Our results suggest that BMP-2 may influence extracellular matrix synthesis and scleral reconstruction, which contributes to the development of human myopia. Further research on BMP-2 and its effects on the alteration of scleral proteoglycan and collagen synthesis and the control of ocular growth in animal models will contribute to the increased understanding of the remodeling of the sclera.
